# Realizing the potential of organoids—an interview with Hans Clevers

**DOI:** 10.1007/s00109-020-02025-3

**Published:** 2021-01-19

**Authors:** Sina Bartfeld

**Affiliations:** Research Centre for Infectious Diseases, Institute for Molecular Infection Biology, Julius Maximilian University of Wuerzburg, Josef Schneider Str. 2/D15, 97080 Wuerzburg, Germany

The past decade has witnessed the development of a powerful new model to study human disease: stem cell–derived organoids. These are 3-dimensional structures recapitulating the architecture of an organ in miniature. Because they can be generated from any patient, organoids offer unprecedented possibilities for medical research, including individualized therapy, toxicology testing, and drug discovery. Given the development of organoids mimicking ever more tissues, including brain, the female reproductive tract, and early embryos, combined with the advance of gene editing technologies, the scientific community and the public are starting to consider the ethical implications of organoid technology. One of the driving forces behind organoid research is Hans Clevers from the Hubrecht Institute in Utrecht, the Netherlands. Here, he discusses the potential of organoids for medicine, the current status of research, and the ethical implications. The interviewer is Sina Bartfeld, a former postdoc of the Clevers lab and now an independent group leader.

SB: The development of organoids proceeded incredibly fast, with less than ten years from discovery to the first use in the clinic. Looking back, what was the first breakthrough?

HC: First, I think it is important to distinguish two different technologies because they use two different types of stem cells. On the one hand, there are the stem cells that can create a whole organism, the pluripotent stem cells, which are either induced pluripotent stem cells or embryonic stem cells. In short iPS or ES cells. On the other hand, what many people do not realize is that there is a second type of stem cell. These so-called adult stem cells constantly regenerate our tissues. For example, the adult stem cells of the intestine will regenerate the inner lining of the intestine, the epithelial cells. Both fields, the iPS/ES field and the adult stem cell field have each developed organoid technologies.

SB: And both fields had their breakthroughs.

HC: I think the one for iPS/ES was the establishment of the first organoids by Yoshiki Sasai in 2008. Before that, people who used ES and iPS cells just wanted to make one cell type, like simply more stem cells, or beta cells, or liver cells, or neurons. Sasai was the first to notice that if you first allow the pluripotent stem cells to form clusters of cells, so-called embryoid bodies, you get more than that, you get structures. He did not yet use the word organoid, but he described that you get anatomical representation [1]. This initiated the ES/iPS organoid field. Our discovery that small intestinal stem cells can build mini-guts then kickstarted the adult stem cell-derived organoids field [2].

SB: Can you remember when you realized the medical potential of organoids?

HC: I think immediately when I saw them. Toshi, Toshiro Sato, who was then a postdoc in my lab, grew them in culture but had not told anybody. I asked, „How is it going, are you growing gut stem cells yet?“. And he said, „Yeah, they are here“. And he showed them to me. The moment I saw them I realized: well apparently you can expand primary tissue and it is not just a lump of stem cells, which is what we thought we would get, but it is really a small organ.

SB: You expected a lump of cells because this is what people had seen in the iPS/ES field?

HC: Yes, in iPS and ES, this is what people did back then, start from one and make a million. Just pure stem cells without any organ-like structure, like a lump of cells. So, our intention was to do the same with adult stem cells. But this is not what they did, the cells organized themselves into these mini guts. When you see them growing, particularly the mouse ones, their vitality is striking. This was very surprising. And when Toshi then also saw that all the cell types of the intestinal epithelium were present in the mini-guts, we realized that if this could be transferred to human stem cells, which Toshi went on to do, you can do everything that people now do in mice in human cells. And you can do it for individual patients. In a personalized fashion. This was not very visionary, I think anybody who saw that experiment would have immediately realized: regenerative medicine, diagnostics, basic science - this could all be done in these organoids.

SB: I remember one lab meeting when you came back from a big medical conference and told us, your group, that you realized that there was an immense need for better models for pre-clinical testing.

HC: Initially we focused on regenerative medicine – we thought this technology’s first use was to build and repair organs. But very soon – and it was all of a sudden - we switched from being a regenerative lab into building disease models and predictive models. We saw that regenerative medicine was going to take ten years, and this was not where we would make great contributions as an academic lab – so we switched to disease modelling.

SB: Which was extremely productive. The prime example is cystic fibrosis, or CF for short, where organoids predict whether or not a patient will respond to a drug.

HC: For CF it is now an established, integral part of the Dutch health care system. I think this is the first time that organoids have formally become part of a healthcare system. CF is a hereditary disease and in Holland, if you had the one most common mutation, you received a particular new drug.

SB: Why only when you had the most common mutation?

HC: Drug development and clinical testing is extremely expensive, so the companies had focused on the most common mutation. The rare mutations had not been included in the clinical testing.

SB: So, it was unclear whether or not the drug would work for these patients. And a test was necessary that would determine whether a particular patient would benefit from the treatment?

HC: And the patient-derived organoids allow such a test [3]. Nowadays, if you have one of the many rare mutations, if your organoids test positively, you also get the drug and reimbursement from the Dutch health insurance. That went very fast. The reasons for that were manifold, but the most important reason was that there was no drug for these patients. When we found that our organoids could predict if a patient would respond, we could then actually give the drug to that patient, because there was no other drug. There are not many other hereditary diseases where this is the case. But currently there is a huge investment to develop organoid-based individual therapies for cancer [4].

SB: How would that work?

HC: A bit like antibiotic resistance testing for bacteria. If you have a bacterial infection, your bacteria are cultured, exposed to a number of antibiotics and then the best one is picked for you. And here, you would grow the cancer cells as organoids, expose them to a number of cancer drugs and select the best drug for the patient based on the response of the organoids. At the moment, this is developing very fast. I know of maybe 30 or 40 trials that are ongoing, observational clinical trials.

SB: How is such a trial set up?

HC: In cancer, there are always protocols and guidelines, so there you cannot say “your organoid tells us that you should not take this drug, you should take something else” – that is not allowed. And it is also not wise. The evidence has to be built up in a statistical fashion, which is currently happening in many places. In these trials, you make organoids from each patient, test the drug response on the organoids, and just observe the outcome of the patient’s standard treatment. And then see whether you would have predicted that response using organoids. The study by Giorgios Vlachogiannis in London, published in 2018 in Science [5], did this very well. Then in 2019 there were several papers that also involved my group [6]. One of them, a study led by Emile Voest in Amsterdam, showed in a trial setting that organoids are highly predictive [7]. Actually, they are surprisingly highly predictive, maybe because these studies are small and the data are looked at extensively. This is very promising.

SB: Is there already an intervention trial?

HC: I know that people want to do this. But then you have to wait until a patient is out of all the guideline protocols and these are of course very difficult-to-treat patients. There needs to be a lot more evidence before you would be allowed to not give a proven therapy to a patient and replace it with something that you think is better.

SB: And which evidence?

HC: Well usually when a new drug is being introduced, this is as an add-on. In such a trial, patients are treated with the standard, but then the new drug is added to the mix. Then you observe which patient does better. In this way, you become more certain. And then maybe you can leave out some of the original drugs. This is the typical development.

SB: So, these are CF and cancer. For which other diseases could organoids be useful?

HC: For example, a lab in Hong Kong used airway organoids to score if new influenza strains are dangerous or not. The new global epidemic viruses are often reassortments from bird flu or from pig flu with human flu virus and they often come from southeast Asia and China. Currently, when new strains appear, they are being tested on slices of human lung to see whether they infect them. These are usually tissue slices from lung cancer patients, and they cannot be kept for very long, it is an awkward system. Two studies we were involved in, both published 2018, showed that human airway organoids can actually discriminate between a virus that is bad for humans and a virus that is not bad for humans [8]. And in a standardized fashion, because the human airway organoids grow forever, so you can have standardized lines. I think it is an important application.

SB: The other big field for organoid application is safety toxicology.

HC: There is a lot of interest in that now. The basic idea: Rather than using animals just use a panel of organoids. Such as liver organoids from ten donors, plus kidney organoids from ten donors, plus small intestinal organoids from ten donors. Currently, new drugs are tested in animals, but animal experiments are not as predictive as you might think. A lot of drugs fail in phase one clinical trials because of toxicity, because it was not picked up in animals.

**Fig. 1:**
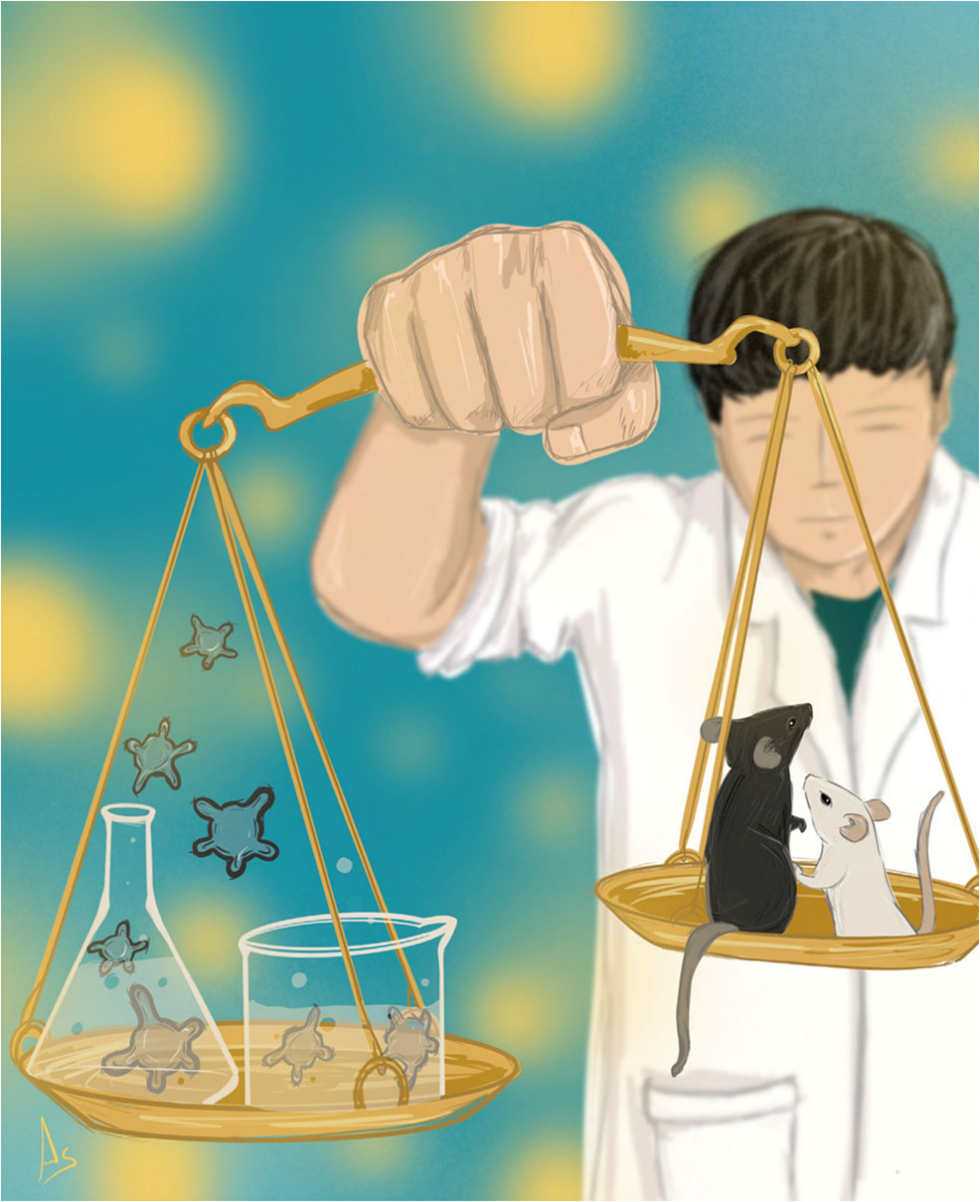
Organoids model specific aspects of organs and are more and more widely used as disease models. Nevertheless, the technology has limitations: “No collection of organoids is equivalent to a mouse or an entire human being”, says Hans Clevers. Yet, organoids have the potential to reduce and replace some of the animal testing, especially in toxicology studies. Clevers: “One important reason is that many diseases are not modeled well at all in mice. Another reason is the ethics, if you can avoid animal experimentation, you should do it”. Graphic by Andreia Batista Rocha

SB: This is a big ethical problem as well as a financial issue for the pharmaceutical industry (Fig. 1).

HC: I talk to the head of safety of a big pharmaceutical company quite a lot and he wants to get rid of animal experiments entirely. He says we just need human models. But the human models will be noisy, much noisier than animals.

SB: Why is that?

HC: If you just take organoids from ten people, you already get quite different results. This is of course because the individuals have a diverse genetic background and environment – if you take ten genetically identical mice that lived under standardized laboratory conditions, you get the exact same response. Which is why the toxicologists like the animals: The curves are nice. But it does not describe reality. Therefore, for safety testing, pharmaceutical companies are heavily interested in replacing animal models with organoids or organs-on-a-chip. This would be very desirable. But again, new tests always have to prove themselves.

SB: How are you contributing to this?

HC: We are doing this in the Hubrecht Organoid Technology Foundation, short HUB, a not-for-profit company founded by the Hubrecht Institute, the Royal Netherlands Academy of Arts and Sciences, KNAW, and the University Medical Center Utrecht. There are a series of examples from pharmaceutical companies where a drug has failed in the phase one clinical trial. Which means the drugs were not toxic in any of the animals, but toxic in humans. The HUB has received some panels of drugs that contain some of these phase-one-failures. And the organoids picked them out right away. This is now being published. I think this is a field that will receive a lot of attention in the coming years.

SB: This sounds as if organoids really could replace animals for toxicology testing entirely.

HC: Yes, this potential is very clear. There are several reasons. One important reason is that many diseases are not modeled well at all in mice. Another reason is the ethics, if you can avoid animal experimentation, you should do it.

SB: Despite all the great aspects of organoid technology, there are also some limitations. What are the most important ones in your view right now?

HC: The two technologies, iPS/ES and adult stem cell-derived organoids, are very different. The main limitation of our organoid technology, so the one using adult stem cells, is that we only grow epithelial cells. That we do very well. We can start from primary tissue, you do not need pure stem cells to start from, you just need thick tissue containing the stem cells. We can do that for many organs, but not all of the organoids are complete.

SB: In which respect?

HC: For example, in the gut, we see all the cell types, but in the stomach, we still lack the parietal cells [9, 10]. Things need to be improved there. Another limitation of our technology is that we can only grow epithelium, we cannot grow muscle, brain, bone or fat. I am convinced that these tissues have stem cells that probably adhere to different rules and probably need very different conditions but it should also be possible to develop bone organoids and fat organoids from adult stem cells. These stem cells, for example bone marrow stem cells - the stem cells we have known about the longest, cannot be expanded in culture. I am sure this is a technical challenge that can be dealt with but this is one limitation: we can only grow epithelium. Also, we do not have microbes, we do not have immune cells, we do not have blood vessels – they have to be added from the outside.

SB: And the other technology, the iPS and ES cell-based organoids?

HC: They can make organoids from tissues that we cannot, central nervous system [11] in particular, also kidney [12, 13]. For example, the glomerulus of the kidney has no regenerative capacity and cannot be grown as an adult stem cell-derived organoid - probably as a consequence of that, but if you grow kidneys from iPS cells you grow glomeruli because it uses the developmental principle rather than the repair principle. And as Jim Wells in Cincinnati has shown, if you take iPS cells through their journey and then try to turn them into endoderm and into gut, then actually not all the cells will follow the exact path and some stay behind as mesodermal cells and they will make muscle and so they create tissues that are actually more complete [14, 15]. Yet, there is a lot of discussion about the variability of iPS and ES-derived organoids. This is because you take these iPS or ES cells on a long journey to become an organoid, this journey can be three months or something like that. And if there are slight differences at the start, they can be amplified enormously during that journey. For example, if you have ten mini-brains and slice them, they are all different. This is again technical; it will probably get better and people will know better how to specify particular regions of the brain that they would like to create.

SB: But this is only the case for the ES and iPS-based organoids?

HC: I would stress that this does not hold for the adult stem cell-derived organoids. They are pretty homogeneous.

SB: So how close is a brain organoid to a brain?

HC: Even if they are ideal, organoids are always a reductionist abstraction of real life, of a real organ or real body, so people should not think that for example a brain organoid is equivalent to an entire brain. No collection of organoids is equivalent to a mouse or an entire human being.

SB: But they can become a part of the body when transplanted. Which brings us to the ethical implications of organoid technology. What do you think about chimeras, especially brain organoid chimeras: If you transplant a human brain organoid into an animal?

HC: Ethical issues are extremely important to address. But I’m not sure that biologists are better positioned than people in general to form opinions on what is desirable and what should be allowed and what should not be allowed. I think what biologists can do is essentially note all the possibilities that all of a sudden occur when you discover a new technology and then find good ethicists and legal people to go through the process of deciding whether this is something we should be doing or should not be doing.

SB: How do you, personally, feel about it?

HC: To be honest, people have transplanted human brain organoids onto mouse brains and that makes me – as a person – a little uncomfortable. I do not know why. This is not our field of science. This is where ethicists have to step in and help define rules how to go about it. I do not think that this one-millimeter piece of human brain organoid will have a consciousness, but you do not know.

SB: How do you feel about embryoids, small iPS-derived organoids representing the early embryo?

HC: Well that is another one that personally makes me uncomfortable. But this is not as a scientist, because as such that is very interesting. But as a father I guess or as a citizen, I would like to have this extensively discussed, whether we, as a society, want this. Because you actually create life without a sperm and an oocyte. This is not how this world has been designed.

SB: And the female reproductive tract? For example, building a placenta-organoid, meaning tissue that could in principle, one day, support an embryo?

HC: About that, I feel less strongly. In this respect, the discussion about IVF in the seventies is very interesting. People were horrified by this idea. Now in some countries, one in four babies is born by IVF and it is not an issue at all. Different cultures think about this very differently. An artificial uterus is sort of a technical thing, it is not as close to the essence of life as an embryo that did not result from two germ cells would be. Or a mouse with a part-human brain. I think they are different things. And we have to discuss them.

SB: If you could dream: In twenty years, what will be possible?

HC: In twenty years, I think organoids will have replaced animal experimentation in toxicology testing. There will be many more applications to use organoids to predict human responses, either to drugs or for example to infections. And, who knows, in twenty years maybe people will also have started to use organoids as transplantation material, maybe we will be able to re-build a liver or a pancreas with organoids. But there is still quite a way to go for that.
